# Effects of FGFR inhibitors TKI258, BGJ398 and AZD4547 on breast cancer cells in 2D, 3D and tissue explant cultures

**DOI:** 10.1007/s13402-020-00562-0

**Published:** 2020-10-29

**Authors:** T. E. Kähkönen, M. Toriseva, N. Petruk, A.-R. Virta, A. Maher, N. Eigéliené, J. Kaivola, P. Boström, I. Koskivuo, M. Nees, J. M. Tuomela, K. K. Ivaska, P. L. Härkönen

**Affiliations:** 1 University of Turku, Institute of Biomedicine, 20520 Turku, Finland; 2 FICAN West Cancer Centre, 20520 Turku, Finland; 3 Department of Pathology, Turku University Hospital, 20520 Turku, Finland; 4 Department of Plastic and General Surgery, Turku University Hospital, 20520 Turku, Finland; 5 Department of Biomedicine and Molecular Biology II, Uniwersytet Medyczny w Lublinie, 20-095 Lublin, Poland

**Keywords:** Breast cancer, Fibroblast growth factor receptor, Fibroblast growth factor receptor inhibitor, 2D/3D cell culture, Explant culture, Radio-sensitization

## Abstract

**Purpose:**

Fibroblast growth factor receptors (FGFR) and pathways are important players in breast cancer (BC) development. They are commonly altered, and BCs exhibiting FGFR gene amplification are currently being studied for drug development. Here, we aimed to compare the effects of three FGFR inhibitors (FGFRis), i.e., non-selective TKI258 and selective BGJ398 and AZD4547, on different BC-derived cell lines (BCCs) and primary tissues.

**Methods:**

The human BCCs MCF-7 and MDA-MB-231(SA) (wild-type *FGFR*) and MFM223 (amplified *FGFR1* and *FGFR2*) were analyzed for FGFR expression using qRT-PCR, and the effects of FGFRis on FGFR signaling by Western blotting. The effects of FGFRis on proliferation, viability, migration and invasion of BCCs were assessed in 2D cultures using live-cell imaging, and in 3D cultures using phenotypic analysis of organoids. To study radio-sensitization, FGFRi treatment was combined with irradiation. Patient-derived BC samples were treated with FGFRis in explant cultures and immunostained for Ki67 and cleaved caspase 3.

**Results:**

We found that all FGFRis tested decreased the growth and viability of BC cells in 2D and 3D cultures. BGJ398 and AZD4547 were found to be potent at low concentrations in *FGFR*-amplified MFM233 cells, whereas higher concentrations were required in non-amplified MCF7 and MDA-MB-231(SA) cells. TKI258 inhibited the migration and invasion, whereas BGJ398 and AZD4547 only inhibited the invasion of MDA-MB-231(SA) cells. FGFRi treatment of MCF7 and MFM223 cells enhanced the inhibitory effect of radiotherapy, but this effect was not observed in MDA-MB-231(SA) cells. FGFRi-treated primary BC explants with moderate FGFR levels showed a tendency towards decreased proliferation and increased apoptosis.

**Conclusions:**

Our results indicate that, besides targeting *FGFR*-amplified BCs with selective FGFRis, also BCs without FGFR amplification/activation may benefit from FGFRi-treatment. Combination with other treatment modalities, such as radiotherapy, may allow the use of FGFRis at relatively low concentrations and, thereby, contribute to better BC treatment outcomes.

**Supplementary Information:**

The online version of this article (10.1007/s13402-020-00562-0) contains supplementary material, which is available to authorized users.

## Introduction

Genetic alterations in fibroblast growth factor receptors (FGFRs), including amplifications, single nucleotide polymorphisms (SNPs) and fusions [1, 2], as well as FGFR ligands (fibroblast growth factors, FGFs) and downstream signaling pathways [3–5] have been found to correlate with breast cancer (BC) development and a poor patient survival. FGFR1–4 are encoded by different genes [6]. FGFRs represent receptor tyrosine kinases that are activated by dimerization upon FGF ligand binding and transphosphorylation of intracellular tyrosine residues. FGF1 and FGF2 can activate all FGFRs, whereas other FGFs can bind receptor isoforms in a ligand-specific manner [4, 5, 7, 8]. FGFR activation elicits downstream signaling by phosphorylating fibroblast growth factor receptor substrate 2 (FRS2), a primary mediator, which leads to activation of MAPK-ERK1/2, PI3K-AKT and PLCγ/MAPK as the main intracellular pathways [4, 7–11]. FGFRs have been reported to regulate BC cell proliferation, apoptosis, survival, migration and angiogenesis via these pathways [4, 7–11].

Among the various possible genetic alterations, *FGFR1* amplification has been observed in 8–15% of BCs [2, 4, 7, 9, 12–15]. *FGFR1* overexpression due to gene amplification or other genetic alterations has been identified as an independent predictor of poor survival, and to contribute to resistance to endocrine therapy [2, 4, 5, 9, 13, 15]. Certain SNPs in the *FGFR2* gene have been found to be associated with BC risk [4, 10] and with the transcription factors Oct1 and Runx2, which can cluster with the estrogen receptor (ER) binding site. Not surprisingly, these SNPs are commonly found in ER-positive BCs [4, 7]. Additionally, *FGFR2* amplifications have been found in triple negative BCs (TNBCs) [2, 4, 12, 16] and in familial *BRCA2*-mutated BCs [7], and to correlate with increased BC cell survival [4]. An increased incidence of BC in patients carrying germline *FGFR3* mutations has turned this gene into a susceptibility factor [4]. The *FGFR4* gene has been found to harbor certain SNPs in hormone receptor-positive BCs [2], and these SNPs have been linked to a poor prognosis [4]. Additionally, *FGFR* gene fusions have been detected [5, 7, 17].

Based on these observations, FGFRs are considered as attractive therapeutic targets, and several FGFR inhibiting compounds are currently being tested in preclinical and clinical studies for the treatment of cancers with *FGFR* amplifications [5]. These compounds include antibodies, ligand traps, and small-molecule tyrosine kinase inhibitors (TKIs) that can covalently bind to the ATP-binding pockets of FGFRs and, thereby, inhibit FGFR activation and intracellular signaling [4, 5, 18, 19]. In recent years, several FGFR tyrosine kinase inhibitors (FGFRis) have been developed. These inhibitors were designed to target activating mutations in FGFRs and include FGFR non- selective multi-kinase inhibitors, such as TKI258 (dovitinib), E7080 (lenvatinib) and BIBF-1120 (nintedanib), and selective FGFRis such as BGJ398 (infigratinib), AZD4547, Debio1347 and JNJ-42756493 (erdafitinib) [18, 19]. The non-selective FGFRis can also inhibit, next to FGFRs, other receptor tyrosine kinases, such as VEGFRs and PDGFRs. Besides genetic receptor alterations, the FGF/FGFR pathways can be deranged and activated by several different mechanisms. These mechanisms include increased FGFR or FGF expression associated with disturbed tumor-stroma interactions, which are crucially important for the growth and progression of BC [20]. BCs are commonly heterogeneous in nature containing different cell types.

We hypothesize that several types of BC may benefit from FGF/FGFR inhibition. To test this hypothesis, we examined and compared the effects of three different FGFRis on the growth of three different types of BC-derived cell lines (BCCs). As FGFRis, we chose the non-selective tyrosine kinase inhibitor TKI258 (dovitinib) and two selective FGFRis, BGJ398 and AZD4547, targeting FGFR1, 2 and 3, respectively. As BC models we used *FGFR*-amplified MFM223 cells [15, 21] and non- amplified, well-differentiated MCF7 and more aggressive MDA-MB-231(SA) cells in in vitro 2D and 3D cultures, and primary human BC samples in ex vivo explant cultures.

## Materials and methods

### Cell culture and FGFRis

MCF-7 cells were purchased from the American Type Culture Collection (ATCC) and MFM223 cells from Sigma-Aldrich. MDA-MB-231(SA) cells were a gift from Dr. Guise (University of Texas, USA). MDA-MB-231(SA) is a variant of MDA-MB-231 that effectively forms bone metastases, but does not otherwise markedly differ from the parental cell line [22, 23]. MCF-7 cells were grown in RPMI-1640 medium (Sigma-Aldrich) supplemented with 10% heat-inactivated fetal bovine serum (iFBS, Gibco), GlutaMAX (Gibco), estrogen (10^–9^ M) and insulin (4 μg/ml). MDA-MB-231(SA) cells were grown in 10% iFBS/DMEM (Sigma-Aldrich) supplemented with GlutaMAX and non-essential amino acids (Gibco). MFM223 cells were grown in 10% iFBS/DMEM. The FGFRis TKI258 (S2769), BGJ398 (S2183) and AZD4547 (S2801) were purchased from Selleck Chemicals as DMSO stocks (IC50 values are listed in Supplementary Table S1).

### Quantitative RT-PCR

Cells were harvested in RLT-buffer (Qiagen) after which RNA isolation was carried out using a RNeasy RNA isolation kit (Qiagen) according to manufacturer’s instructions. Purified RNA (1 μg) was converted to cDNA using Oligo-d(T)18 mRNA primers (New England Biolabs) and Maxima RT enzyme according to the manufacturer’s instructions with the addition of dNTPs and a RNAse inhibitor (Thermo Fisher Scientific). cDNA was subjected to quantitative real-time PCR (qRT-PCR) using a DyNAmo HS SYBR Green qPCR kit (Thermo Fisher Scientific) with gene-specific primers (Supplement 2). The samples were run on a Bio-Rad CFX96 qPCR system with an annealing temperature of 60 °C for in total 40 cycles. β-actin was used as a reference gene. The results were analyzed by adjusting the Ct-values to that of β-actin and subsequently comparing the relative expression of receptors within each cell line.

### Western blotting

MCF-7, MDA-MB-231(SA) and MFM223 cells were starved in 1% bovine serum albumin (BSA)/DMEM for 24 h and treated with FGFRis (1 nM to 10 μM) or 0.1% DMSO (control), after which 25 ng/ml FGF2 (R&D Systems) was added 30 min after addition of the inhibitors. After 10 min, the cells were lysed in 5x Sample buffer (0.5 M Tris-HCl, glycerol, 20% SDS, bromophenol blue). Equal amounts of cell lysates were loaded and run on 12% SDS-PAGE gels and transferred to protein absorbing membranes blocked with 5% fat-free milk solution. Next, the membranes were incubated with primary antibodies at 4 °C o/n. The binding of primary and matching secondary antibodies was visualized using a Li-Cor Odyssey® CLx imaging system (Li-Cor) for MFM223 cells and for total ERK1/2 in MDA-MB-231(SA) cells, or an ECL method (GE Healthcare) with detection on Kodak films (Perkin Elmer). Cell lysates were harvested from sub-confluent cell layers in normal growth conditions to detect FGFR protein levels in MCF-7, MDA-MB-231(SA) and MFM223 cells. Protein concentrations were measured using the Bradford assay. Equal protein amounts from each cell line were analyzed as described above, using a Li-Cor Odyssey® CLx imaging system. The antibodies used for Western blotting are listed in Supplementary Table S2.

### FGFRi treatment and proliferation and viability assays

MCF-7, MDA-MB-231(SA) and MFM223 cells were seeded in 96-well plates at densities of 15.000, 5.000 and 10.000 cells/well, respectively. On the next day, the FGFRis TKI258, BGJ398 and AZD4547 were added at final concentrations of 1, 5 and 10 μM for MCF-7 and MDA-MB-231(SA) cells, and 1 nM, 10 nM, 100 nM, 1 μM and 10 μM for MFM223 cells (*n* = 4–10). DMSO (0.1%) was used as control treatment. Starting from the initiation of FGFRi treatment, the cells were imaged using an IncuCyte® ZOOM real-time live-cell imaging system (Essen Bioscience) with a 10x objective and a 2 h imaging interval for 70 h. Confluency was analyzed, and kinetic growth curves were generated using IncuCyte ZOOM imaging software.

After the 70 h culture period with inhibitors, the cells were subjected to viability assessment using alamarBlue (Thermo Fisher Scientific) for proliferation assays and Cell Counting Kit 8 (CCK8, Dojindo Molecular Technologies Inc.) for irradiation sensitizing assays. The cells were incubated with the reagents for 2 h, after which fluorescence (550/580 nm, alamarBlue) or absorbance (450/690 nm, CCK8) were measured using microplate readers (Hidex or Tecan ULTRA).

### Irradiation assays

In the first experimental setup, the cells were seeded into 96-well plates and left to attach for 24 h. Next, the cells were treated with FGFRis for 24 h as described above and subsequently irradiated with a single dose of 4 Gy X-ray (4 mA and 350 kV, MultiRad 350 system, Faxitron) for 2 min. After another 24 h, fresh medium was added to the cultures and cell viability was measured after 48 h. Control cultures were not irradiated. In the second setup, the cells were first irradiated with 4 Gy X- ray and subsequently treated with FGFRis for 24 h. Fresh medium was added to the cells and cell viability was determined after a 48 h incubation using a CCK8 assay as described above.

### Cell migration and invasion assays

The effects of FGFRis on MDA-MB-231(SA) and MFM223 cell migration were studied using an IncuCyte® ZOOM (Essen Bioscience, Sartorius) scratch wound healing assay. Briefly, cells were seeded into 96-well ImageLock plates and grown to 95% confluence. Next, scratch wounds were made in the cell layers using the WoundMaker tool (Essen Bioscience, Sartorius) after which medium containing different concentrations of TKI258, BGJ398 and AZD4547 was added to the cultures. Subsequent wound closure was monitored using real-time imaging (4x objective, 2 h imaging interval) and analyzed using IncuCyte ZOOM software. As an invasive cell line model, MDA-MB-231(SA) cells were also subjected to collagen invasion assessment. To this end, ImageLock plates were first coated with neutralized 2% rat tail type 1 collagen (BD Bioscience) and incubated in 37 °C for 1 h, after which the cells were seeded. On the following day, scratch wounds were made and another layer of collagen was applied to provide a 3D collagen matrix for the cell cultures. Medium containing inhibitors was applied on top. The non-migratory and non-invasive MCF-7 cells were not included in these studies.

### Organotypic 3D cell culture assays

Organotypic 3D cell culture assays were performed as described previously [24]. This cell culture model supports the formation of biologically relevant cell-cell and cell-matrix interactions and the potential formation of well-differentiated organoids [24]. Briefly, MCF-7, MDA-MB-231(SA) and MFM223 cells were seeded as single cells between two layers of Matrigel™ Matrix Growth Factor Reduced (GFR) (BD Biosciences) on 96-well angiogenesis μ-plates (Ibidi GmbH) and, after polymerization at 37 °C, normal cell growth medium was added on top. Next, organoids were allowed to form for 3–4 days and treated with FGFRis for another 6–7 days at different concentrations (in total 10 days in culture). The medium with FGFRis was changed every 2–3 days. At the endpoint, the organoids were imaged using a wide-field phase-contrast microscope (Zeiss Axiovert 200 M with AxioCam MRm camera and a 10x objective for MCF-7 and MDA-MB-231(SA) cells, and Nicon Eclipse Ti with DS-Qi2 camera and a 20x objective for MFM223 cells). Subsequently, the cultures were stained with Calcein-AM and Ethidiumhomodimer-1 (EthD1) dyes (both from Thermo Fisher Scientific) to visualize living and dead cells, respectively. The stained organoids were imaged using a spinning disk confocal microscope (Axiovert 200 M, 5x objective) after which the images were converted to maximum intensity projections using SlideBook6 software (3i Intelligent Imaging Innovations, Inc.). The projections were analyzed using automated morphometric image data analysis software AMIDA [25]. Individual organoids were identified and included in the image segmentation analysis. The resulting quantitative morphometric data derived from the 3D cultures were further visualized and used for treatment comparisons using the R-software environment (www.r-project.org).

### BC explant cultures

Human BC tissue samples were obtained by breast cancer surgery at the Department of Plastic and General Surgery at Turku University Hospital (Turku, Finland) with approval from the Ethics Committee of the Hospital District of Southwestern Finland and written consent from the patients (§279, 9/2001). The patients had not received neoadjuvant treatment prior to surgery. Paired samples from breast tumors and surrounding peritumoral tissues of 10 BC patients were excised and examined by a clinical pathologist and, subsequently, used for the establishment of explant cultures (Supplementary Table S3). In addition, tissue samples were snap-frozen in liquid nitrogen and homogenized into phenol-containing TRIsure™ solution (Bioline, BIO-32033), after which RNA was isolated using a phenol-chloroform extraction method according to the manufacturer’s instructions and FGFR expression was analyzed by qRT-PCR as described under 2.2.

The human breast tissue explant culture system used has previously been optimized [26]. In brief, excess adipose tissue was removed and the tissues were dissected into small pieces of approximately 1–2 mm in size. Two to four pieces of tissue were placed on the top of Spongostan™ dental gelatin sponges (Ethicon) half-merged with medium. The medium used for the explant cultures contained phenol red-free HEPES-buffered DMEM/F12 with L-glutamine (Gibco) supplemented with 10% iFBS, 10 nM hydrocortisone (Sigma-Aldrich), 0.25% insulin-transferrin selenium supplement (Gibco), 0.25 μg/ml Amphotericin B (Gibco) and antibiotics. The cultures were treated with FGFRis, and the medium was changed once during a 7 day culture period. At the endpoint, the explants were fixed in 10% neutral buffered formalin for 24 h and stored in 70% ethanol.

### Immunohistochemistry and quantitative image analysis

Explant cultures were embedded in paraffin and cut into 5 μM sections, after which hematoxylin and eosin (HE) staining was performed using standard methods. For immunohistochemistry, the sections were deparaffinized, blocked with 3% hydrogen peroxide and 10 mM citrate buffer, incubated with antibodies directed against Ki67 (Novocastra, NCL-L-Ki67-MM1) or cleaved caspase 3 (Cell Signaling Technologies, clone 5A1E, #9664) at 4 °C o/n, and with a biotinylated secondary antibody for 1 h at room temperature. Next, a chromogenic HRP-mediated diaminobenzidine staining method was used (Vectastain Elite ABC Kit, Vector Laboratories, USA), after which the samples were counterstained with Mayer’s hematoxylin. The stained sections were scanned using a digital slide scanner (Pannoramic 250 Slide Scanner, 3DHistech). Five representative images from cancerous areas were taken at 40x magnification. The number of stained cells/nuclei was quantified using thresholding within the Image J program, and the total number of tumor cells was calculated manually.

### Statistical analysis

The presentation of data and applied statistical methods are provided in the figure legends. Graph Pad Prism 7 and software R (www.r-project.org) were used for data visualization and statistical analyses.

## Results

### FGFRs 1–4 are expressed in different BC cell lines

**Fig. 1:**
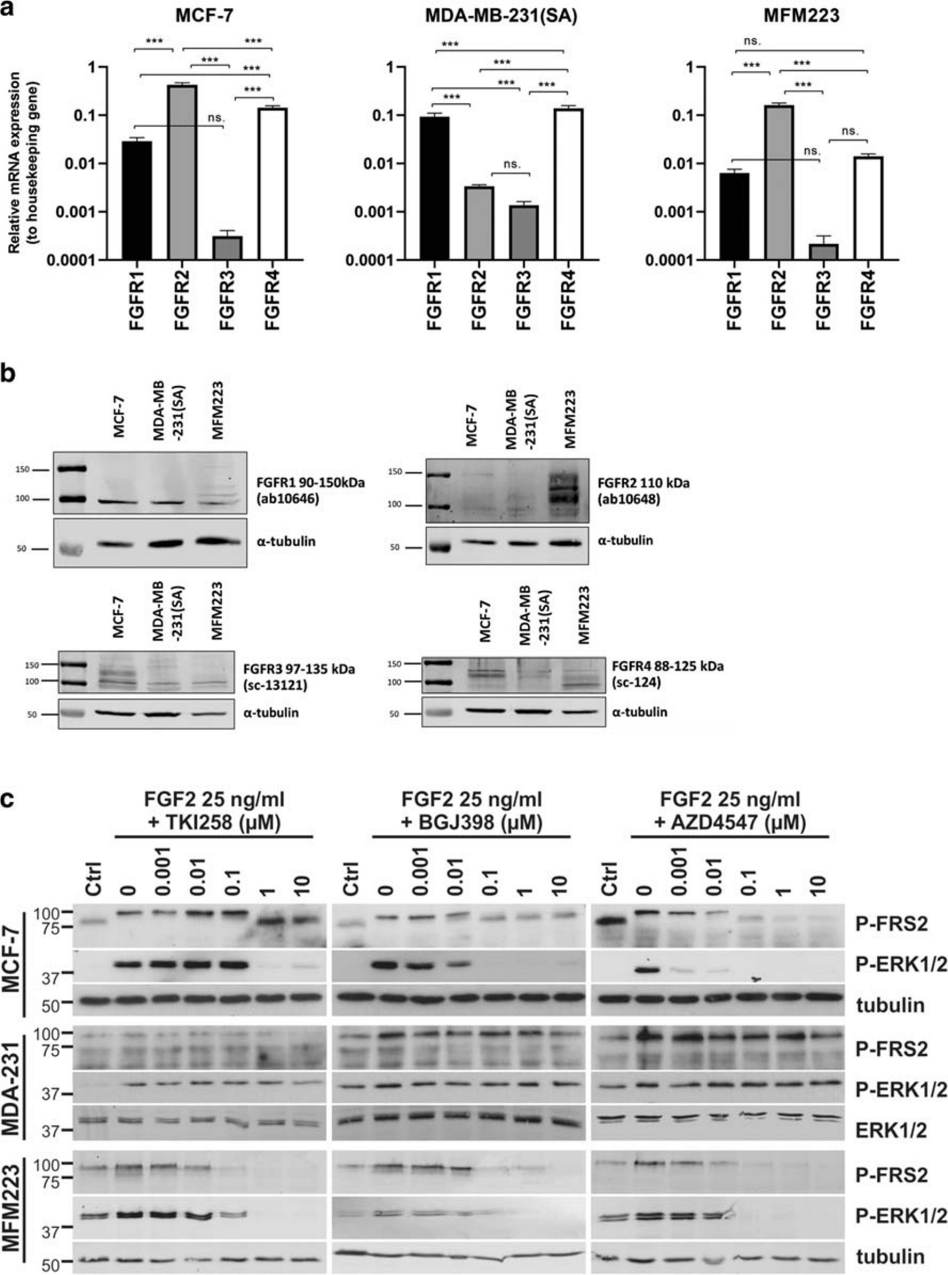
FGFR expression and the effects of FGFRis on FGFR signaling in BC cells. (**a**) mRNA expression of FGFR1–4 in MCF-7, MDA-MB-231(SA) and MFM223 cells analyzed by qRT-PCR. The columns present the relative expression levels compared to the expression level of the housekeeping gene β-actin, calculated separately in each cell line. Statistical significances (one-way ANOVA with Tukey posthoc test) are shown as: ns, * *p* < 0.05, ** *p* < 0.01, *** *p* < 0.001. Note that the scale on the y axis is logarithmic. (**b**) Protein expression of FGFR1, FGFR2, FGFR3 and FGFR4 in MCF-7, MDA-MB-231(SA) and MFM223 cells detected by Western blot analysis. (**c**) Cells were serum starved and treated with FGF-2 for 10 min in the presence or absence of TKI258, BGJ398 and AZD4547 at the indicated concentrations. Cell lysates were analyzed for FRS2 and ERK1/2 phosphorylation levels using Western blotting. Alpha-tubulin and total ERK1/2 were used as a loading controls for MCF-7 and MFM223 cells, and for MDA-MB-231(SA) (abbreviated as MDA-231) cells, respectively

We found that the FGFR expression profiles of MCF-7 and MFM223 cells were similar at the mRNA level, with FGFR2 being most abundantly expressed (Fig. 1a). FGFR1 and FGFR4 mRNAs were also detected at a relatively high level in these cells. In MDA-MB-231(SA) cells, the predominant mRNAs were those for the receptors FGFR1 and FGFR4. The expression of FGFR3 mRNA was low in all three cell lines (Ct values ~5–6 cycles higher than for FGFR1). Despite the reported *FGFR1* and *FGFR2* gene amplifications in MFM223 cells [15, 21], we did not detect higher relative FGFR1 or FGFR2 mRNA expression levels in these cells compared to MCF-7 or MDA-MB-231(SA) cells. Using Western blot analysis, however, we found that the FGFR1 protein expression pattern in MFM223 cells differed from that in the two other cell lines. A relatively high level of FGFR2 expression was detected in MFM223 cells. Different FGFR4 expression levels were detected in all the examined cell lines (Fig. 1b). The protein levels detected by Western blot analysis were, however, not completely in line with the mRNA expression data. Despite a very low FGFR3 mRNA expression, FGFR3 protein was detected in all three cell lines.

### FGFRis decrease FGFR-mediated signaling in BC cells

Serum-starved cells were treated with FGFRis simultaneously with FGF2 to specifically examine the effects of TKI258, BGJ398 and AZD4547 on the FGFR signaling in MCF-7, MDA-MB-231(SA) and MFM223 cells at the molecular level. TKI258 is a wide-spectrum receptor tyrosine kinase inhibitor and generally a weaker inhibitor of FGFR’s than the FGFR selective BGJ398 and AZD4547 inhibitors (Supplementary Table S1). FGFR signaling activity was assessed by analyzing the phosphorylation levels of fibroblast growth factor receptor substrate 2 (FRS2) and its down-stream target mitogen-activated protein kinase ERK1/2. The strongest inhibition of FGF2-stimulated phosphorylation of FRS2 and ERK1/2 by TKI258 was seen in MFM223 cells at 10 nM concentration (Fig. 1c). MCF-7 cells showed a clear inhibition of FRS2 (P-FRS2) and ERK1/2 (P-ERK1/2) phosphorylation at 1 μM TKI258 concentration. The weakest response to TKI258 was detected in MDA-MB-231(SA) cells, which showed reduced P-FRS2 clearly only at 10 μM concentration (Fig. 1c). BGJ398 decreased P-FRS2 and P-ERK1/2 at 100 nM concentration in MFM223 cells. In MCF-7 cells, decreased P-ERK1/2 was detected even at 10 nM concentration (Fig. 1c). Again, MDA-MB-231(SA) cells showed the weakest response with decreased P-FRS2 only at 10 μM BGJ398 concentration (Fig. 1c). Of the three FGFRis, AZD4547 exhibited the strongest inhibitory effect on FGF-2 induced FGFR signaling. Interestingly, the strongest effect was detected in MCF-7 cells with clearly decreased P-ERK1/2 and P-FRS2 levels at 1 nM AZD4547 concentration. AZD4547 decreased P-FRS2 and P-ERK1/2 at 10 nM and 100 nM concentrations, respectively, in MFM223 cells. However, in MDA-MB-231(SA) cells, a slight reduction in P-FRS2 was seen only at 10 μM AZD4547 concentration. MDA-MB-231(SA) cells showed hardly any effect on ERK1/2 phosphorylation with TKI258, BGJ398 or AZD4547, suggesting constitutively active MAPK signaling in these cells not specifically dependent on FGF-2-induced FGFR signaling.

### FGFRis dose-dependently decrease BC cell proliferation and viability in 2D cultures

**Fig. 2:**
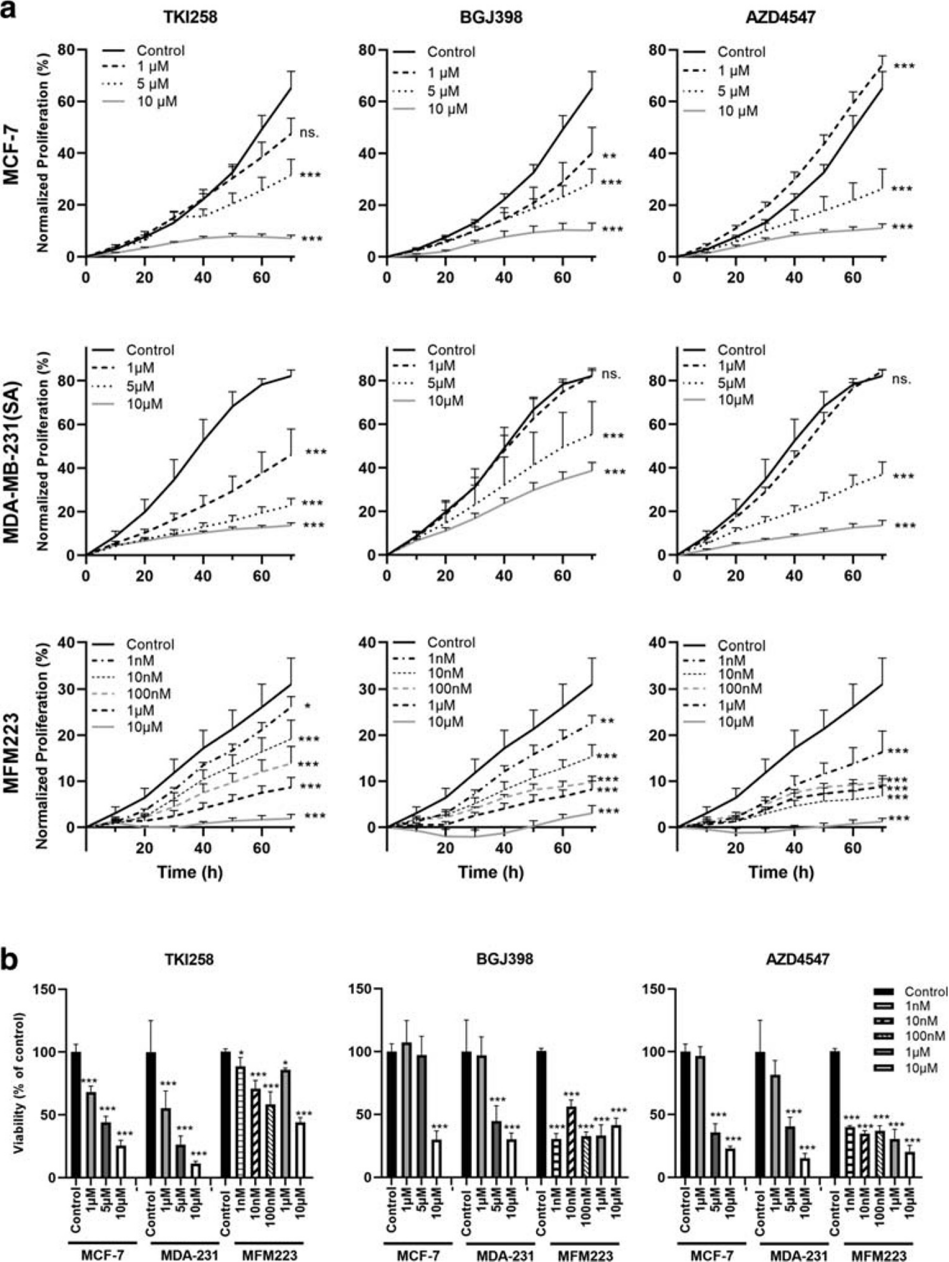
FGFRis decrease the proliferation of BC cells. (**a**) Proliferation of MCF-7, MDA- MB-231(SA) and MFM223 cells studied by measuring cell confluence using the IncuCyte ZOOM real-time imaging platform. One day after seeding, cells were treated with TKI258, BGJ398 or AZD4547 at the indicated concentrations and monitored up to 70 h. Confluence (%, mean ± SD, *n* = 8) was normalized to the corresponding value at 0 h (start of imaging). Statistical significances were analyzed for the area under the curve (AUC) over time and are shown compared to the control group (* *p* < 0.05, ** *p* < 0.01, *** *p* < 0.001). (**b**) Cells were treated as described above and viability was determined at the 70 h point using alamarBlue reagent. The columns represent the relative cell viabilities after 70 h FGFRi treatment compared to the control in each cell line (%, mean ± SD, *n* = 4–10). Statistical significances were determined using one-way ANOVA and adjusted for multiple comparison using Dunnett’s test, with the control group as reference. (* *p* < 0.05, ** *p* < 0.01, *** *p* < 0.001)

We found that TKI258 decreased the proliferation of MCF-7 cells at 5 μM, of MDA-MB-231(SA) cells at 1 μM and of MFM223 cells at 1 nM concentrations (*p* < 0.001, Fig. 2a). BGJ398 decreased the proliferation of MCF-7 cells at 1 μM concentration (Fig. 2a). In MDA-MB-231(SA) cells, no clear dose-response was observed and decreased proliferation was seen at 5 μM concentration (Fig. 2a).

MFM223 cells were more sensitive to BGJ398, and decreased proliferation was seen even at 1 nM concentration. ADZ4547 decreased the proliferation of MCF-7 and MDA-MB-231(SA) cells at 5 μM concentration and in MFM223 cells at 1 nM concentration (Fig. 2a). Overall, MFM223 cells were found to be most sensitive to FGFR inhibition (Fig. 2a). Treatment of the cells with high FGFRi concentrations resulted in strong growth inhibition (Fig. 2a). Moreover, TKI258 resulted in cytoplasmic swelling of MCF-7 cells even at 1 μM concentration, although no visible cell death was observed (cell rounding and detachment). Some morphological signs of FGFRi induced cell death were observed in MCF-7 and MFM223 cells, but not in MDA-MB-231(SA) cells, upon 70 h of treatment at the highest concentrations of FGFRis (Supplementary Fig. S1).

In addition to the image-based, real-time proliferation analysis described above, we used a biochemical cell viability assay (alamarBlue) to detect the amounts of viable cells in the cultures after 70 h FGFRi treatment. In accordance with the proliferation assay, we found that TKI258 decreased the number of viable cells in a concentration-dependent manner, compared to the untreated controls in all the cell lines (Fig. 2b). Similarly, a decreased viability was detected using BGJ398 and AZD4547, which again showed a clear accordance with the real-time proliferation analysis (Fig. 2b). The decreased viability observed likely reflects decreased cell proliferation, because few obvious morphological signs of cytotoxicity were observed (Supplementary Fig. S1). Indeed, after culturing the cells for 70 h in the presence of FGFRis, cell viability remained at about 20% or higher in all treated cells compared to the respective  measurements  at 0 h timepoint even at the highest concentrations (Fig. 2b). This indicates that there were viable cells in all experimental conditions and suggests that the FGFRis did not cause major apoptosis or cytotoxicity in these experiments. Together these results indicate that the studied FGFRis inhibit the proliferation of MCF-7, MDA-MB-231(SA) and MFM223 cells in a concentration-dependent manner.

Because rather high concentrations of FGFRis were needed to obtain growth inhibition of BC cells without inducing major cell death, we next explored whether a combination of FGFRi and irradiation could have additive effects on these cells. We found that irradiation of the BC cells with a suboptimal dose of 4 Gy of X-ray decreased their viability to 50–75% (Supplementary Fig. S2). When MFM223 cells were pre-treated with 1 μM of TKI258 and BGJ398 for 24 h, a small but non- significant decrease was observed. Also in MCF7 or MDA-MB-231(SA) cells pretreatment did not sensitize the cells to irradiation. In contrast, when the cells were first irradiated and then treated with 10 nM or 1 μM TKI258 or BGJ398 or AZD4547, a more pronounced, statistically significant decrease in viability was observed. Corresponding irradiation potentiating responses were also observed in MCF7 cells at 5 μM TKI258 or BCJ398 and in AZD4547 cells, but not in MDA-MB-231(SA) cells.

### FGFRis decrease BC cell growth and increase BC cell death in 3D cultures

**Fig. 3:**
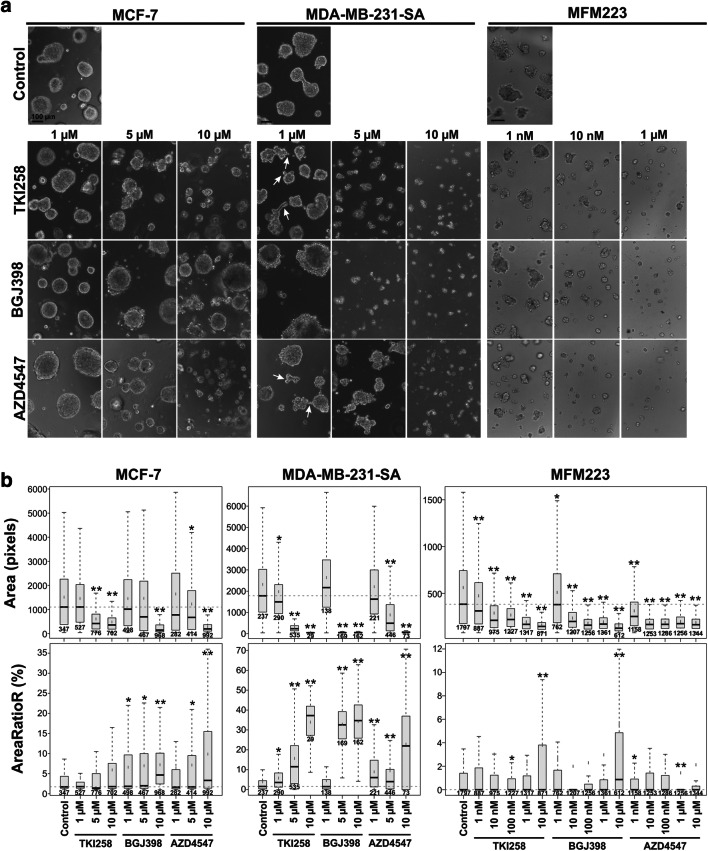
FGFRis affect BC cell organoid formation in an organotypic 3D culture model. MCF-7 and MDA-MB-231(SA) cells, and MFM223 cells were precultured in Matrigel for 3 or 4 days, respectively, and then treated with TKI258, BGJ398 or AZD4547 for 7 days and 6 days, respectively, at the indicated concentrations. (**a**) Representative phase-contrast images of cultures at the endpoint of the treatment. Magnification: 10x for MCF-7 and MDA-MB-231(SA) cells and 20x for MFM223 cells. Scale bar: 100 μm. (**b**) At the endpoint, cell organoids were stained with Calcein AM and EthD-1, imaged, and subjected to phenotypic analysis using AMIDA software. Object size (Area), reflecting the growth response and the overall viability of the cells, and the relative amount of red signal in an object (AreaRatioR), indicating cell death, are presented. Box-and-whisker plots highlight the medians (black horizontal line), means, 1.5 IQR and *p*-values (Bonferroni-corrected *t*-tests, * *p* < 0.05, ** *p* < 0.001), and the number of objects included in the analysis

It has been suggested that responses to drug treatment *in vitro* can be affected by tissue-like microenvironments. It has been shown, for example, that a 3D microenvironment can sensitize MDA-MB-231 cells to MEK-inhibitors [27]. We used an organotypic 3D cell culture model, which displays physiologically relevant cell-cell and cell-matrix interactions and better recapitulates human cancer histology compared to conventional 2D cultures [24] to investigate the functional responses of MCF-7, MDA-MB-231(SA) and MFM223 cells to FGFRis. MCF-7 and MDA-MB-231(SA) cells were allowed to establish small organoids for three days, after which the cultures were treated with FGFRis for 7 days. MFM223 cells were precultured for four days prior to a 6 d FGFRi treatment. At the endpoint, phenotypic analyses were performed to quantify the organoid sizes and cell death rates in these multi-organoid cultures [25]. All the inhibitors decreased the size of organoids as shown by phase-contrast microscopic images at day 10 of culture (Fig. 3a) and by quantitative phenotypic data (Fig. 3b, Area; Supplementary Fig. S3). TKI258 decreased MCF-7 organoid sizes at 5 μM and MDA-MB-231(SA) organoid sizes at 1 μM concentrations (Fig. 3a and b, Area). MFM223 organoids were highly sensitive to TKI258, and decreased organoid sizes were observed at 1 nM concentration. BGJ398 decreased the organoid sizes of MCF-7 cells at 10 μM, MDA-MB-231(SA) cells at 5 μM, and MFM223 cells at 1 nM concentrations (Fig. 3a and b, Area). AZD4547 decreased MCF-7 and MDA-MB-231(SA) organoid sizes at 5 μM concentration, and in MFM223 cells decreased organoid sizes were observed at 1 nM concentration (Fig. 3a and b, Area). In the current model, in which FGFRis were added to cells in the early phase of culture, the organoid sizes primarily reflect decreased growth, but also cell death. The FGFRi effects on organoid sizes were well in line with the inhibition of proliferation in 2D cultures (Fig. 2a).

In contrast to the 2D cultures, increased cell death was observed in the 3D cultures. TKI258 increased the proportion of dead cells in MDA-MB-231(SA) organoids at 1 μM and higher concentrations, but no significant effects were observed in MCF-7 organoids (Fig. 3a and b, AreaRatioR; Supplementary Fig. S3). BGJ398 increased cell death at 1 μM concentration in MCF-7 organoids and at 5 μM concentration in MDA-MB-231(SA) organoids (Fig. 3b, AreaRatioR). AZD4547 increased cell death in MCF-7 organoids at 5 μM and in MDA-MB-231(SA) organoids at 1 μM concentration (Fig. 3b, AreaRatioR). Interestingly, increased cell death was rarely observed in MFM223 organoids, suggesting milder cytotoxic effects despite a markedly longer treatment time compared to 2D cultures.

Untreated MCF-7 and MDA-MB-231(SA) cells formed well-differentiated round organoids, whereas MFM223 organoids were smaller and more irregular in shape (Fig. 3a). In addition, some treatment-induced changes were observed (Fig. 3a). Particularly in MDA-MB-231(SA) organoids treated with low concentrations of FGFRis, a more irregular shape was observed with increased multicellular protrusions, which were not associated with cell death (Fig. 3a, arrows).

### FGFRis differentially regulate the migration and invasion of BC cells

**Fig. 4:**
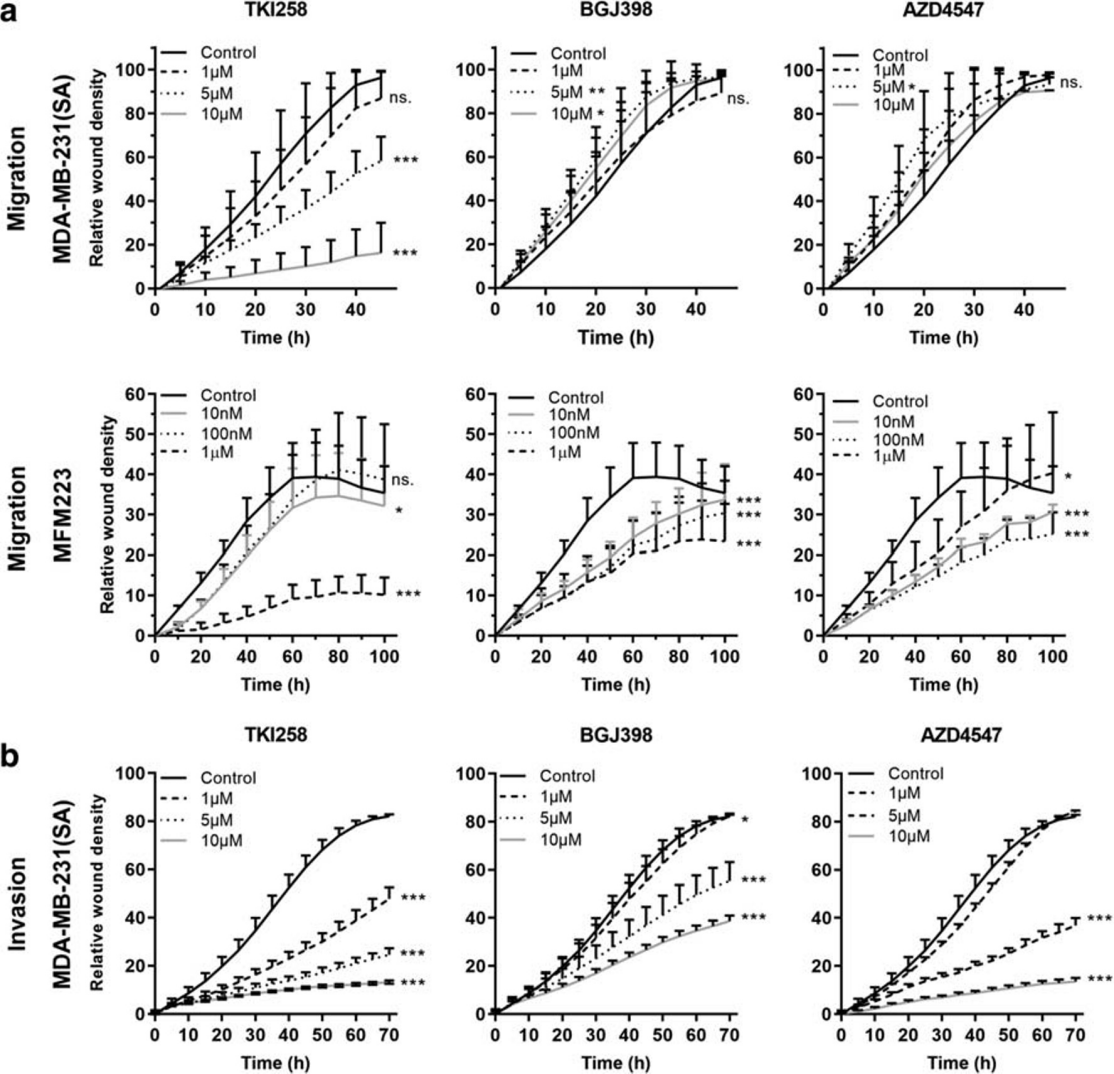
FGFRis decrease migration and invasion of BC cells. (**a**) MDA-MB-231(SA) and MFM223 cells were subjected to scratch wound migration assays in the presence of FGFRis at the indicated concentrations. The cultures were imaged using IncuCyte ZOOM for 48 h (MDA-MB-231(SA) cells) or 96 h (MFM223 cells). The relative wound closure (mean ± SD, *n* = 8) is presented at the indicated time points. (**b**) MDA-MB-231(SA) cells were used in an invasion assay, where the cells were covered with collagen type I matrix. The cultures were imaged using IncuCyte ZOOM for 72 h. The relative invasion (mean ± SD, *n* = 8) is presented for each treatment group. Statistical significances were analyzed for the area under the curve (AUC) over time and are shown for the comparisons to the control groups (* *p* < 0.05, ** *p* < 0.01, *** *p* < 0.001). Note that the inhibitor concentrations used for MDA-MB-231(SA) and MFM223 cells are different

Using the IncuCyte scratch wound assay, we found that TKI258 decreased the migration of MDA- MB-231(SA) cells on plastic at 5 μM concentration (Fig. 4a). In MFM223 cells, a decreased migration was observed at 1 μM concentration, but no dose-dependency was observed (Fig. 4a). BGJ398 and AZD4547 had no effect on the migration of MDA-MB-231(SA) cells, but they decreased the migration of MFM223 cells at 10 nM concentration (Fig. 4a). All the inhibitors decreased invasion of MDA-MB-231(SA) cells through collagen I matrix at 1 μM concentration (Fig. 4b).

### Human BC tissue explants respond to FGFRis

**Fig. 5:**
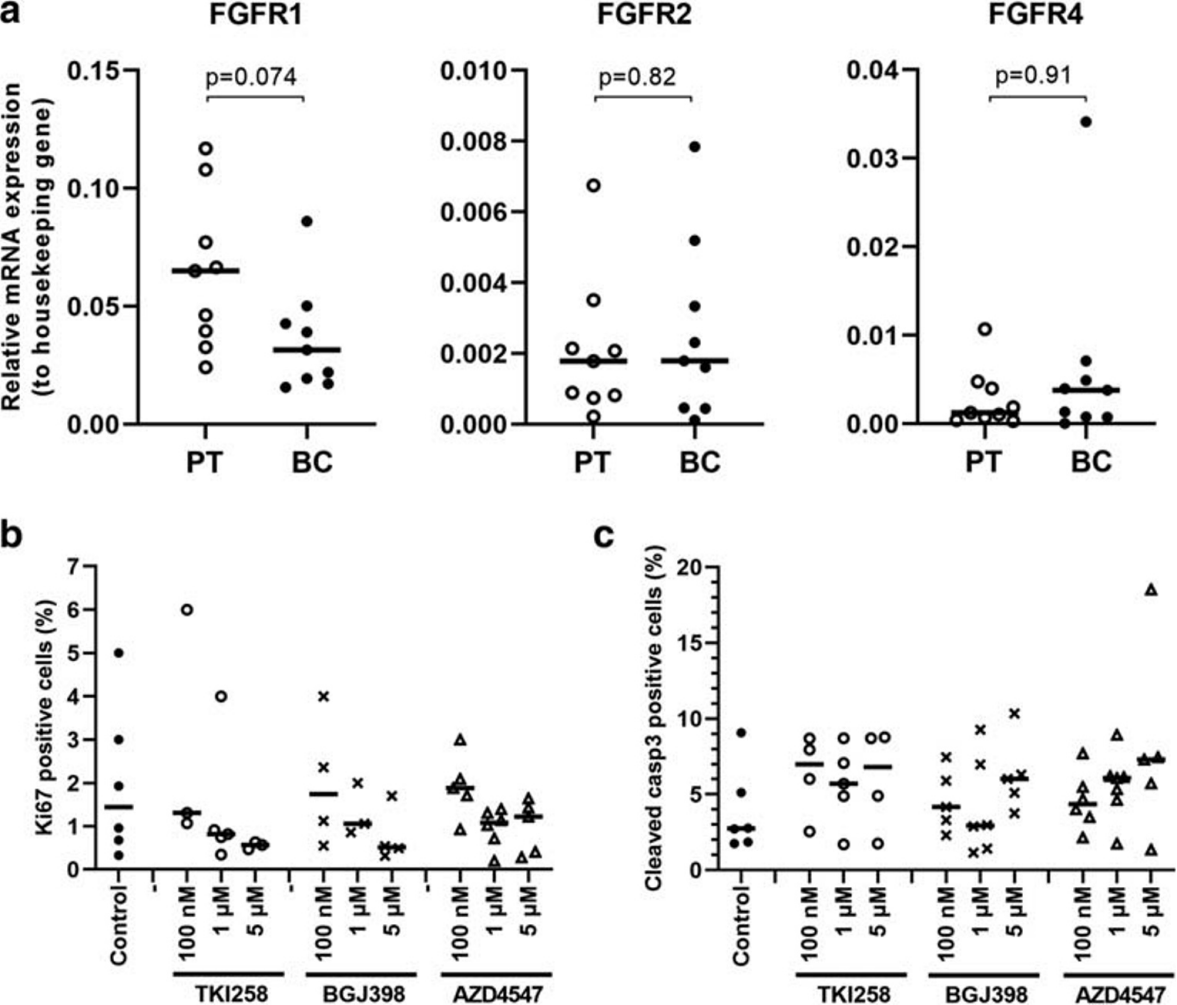
Analysis of human BC ex vivo cultures treated with FGFRis. (**a**) FGFR mRNA expression in human BC tissue samples and the corresponding peritumoral samples (PT) analyzed by qRT-PCR (*n* = 9). The expression is presented relative to that of the housekeeping gene β-actin in each sample, and the horizontal lines represent the median values in each group. The quality of the extracted mRNA was poor in one sample (ID 3), and thus is omitted from the figure. *P* values for the differences between paired BC and PT samples are shown (Wilcoxon signed-rank test). Note that the scale is different for the various FGFRs. (**b**) Relative numbers (%) of cells positive for Ki67. (**c**) Relative numbers (%) of cells positive for cleaved caspase 3. For quantification of immunohistochemistry, the total cell number (nuclei, on average one thousand cells), and the total number of positive cells were used to calculate the percentage of positive cells. Data are presented from all the samples with positive staining (*n* = 3–6 per treatment) and the horizontal line represents the median. There were no statistically significant differences in the relative numbers of cells positive for Ki67 (*p* = 0.22) or cleaved caspase 3 (*p* = 0.75) between the treated explants compared to control explants (Kruskall-Wallis test, followed by Dunn’s multiple comparison test)

In tissue samples obtained from consecutive surgeries of primary human breast tumors, FGFR1 was most abundantly expressed at the mRNA level, followed by FGFR4 and FGFR2 (Fig. 5a). The level of FGFR3 mRNA was too low to be reliably detected (high Ct values). The FGFR expression patterns varied between the patients. On average, the expression of FGFR1 mRNA appeared to be lower than that of FGFR4 in tumor tissue compared to peritumoral tissue. FGFR2 was similarly expressed in both tissues. Due to variations in peritumoral samples between individual patients, the relative expression levels are not comparable.

The integrity and morphological features of the explants remained through the 7 d treatment periods with FGFRis (data not shown). Treatments at 100 nM, 1 and 5 μM FGFRi concentrations suggested a trend towards a decrease in the relative numbers of Ki67-positive tumor cells (Fig. 5b) and an increase in the relative numbers of apoptotic cells (Fig. 5c). However, the small amounts of sample tissues from each patient, the intrinsic heterogeneity of BC tissues and the relatively low number of patient samples limit a reliable generality of these results in this pilot study.

## Discussion

The effects and treatment options of FGFRis are widely studied in different cancers, including BC [18, 19]. However, to the best of our knowledge, this is the first study comparing three different FGFRis in various experimental models for human BC. One of the compounds, TKI258, is a non-selective FGFRi that also inhibits other receptor tyrosine kinases such as FLT3, C-KIT, VEGFRs, CSF1R and PDGFRs, whereas the other two, BGJ398 and AZD4547, represent more selective FGFRis. All these inhibitors are currently being evaluated in clinical trials for different indications, including BC [19, 28].

Our results showed that the main effect of the FGFRis was to decrease BC cell proliferation. This was observed both in 2D and 3D cultures, and also suggested by ex vivo explant cultures. *FGFR*-amplified MFM223 cells expressing particularly FGFR2 at a high level were most sensitive to the selective FGFRis BGJ398 and AZD4547, which decreased their proliferation at nanomolar concentrations, whereas higher concentrations were needed to inhibit the proliferation of MCF-7 and MDA-MB-231(SA) cells, which express FGFRs at a moderate and a rather low level, respectively. Based on our results on FGFR-mediated signaling and proliferation, we conclude that MCF-7 cells are sensitive to inhibition of the FGFR pathway, although they do not show major genetic *FGFR* alterations. MDA-MB-231(SA) cells appeared to be more dependent on other growth factor receptor-mediated pathways. These cells were most sensitive to TKI258 treatment which, besides FGFRs, also inhibits other tyrosine-kinase receptors (Supplementary Table S1). No inhibition of P-ERK1/2 signaling was observed in MDA-MB-231(SA) cells, and phosphorylation of FRS2 was only inhibited at a high (10 μM) concentration. This suggests that the ERK pathway in these cells is mainly induced and maintained by signaling cascades other than that of FGFR. In MCF-7 and MFM223 cells, but not in MDA-MB-231(SA) cells, decreased proliferation was found to be associated with reduced P-ERK1/2 levels, providing one mechanistic explanation. Other pathways such as the PLCγ pathway, also regulate proliferation, and it is possible that one or more of these pathways was inhibited in MDA-MB-231 cells as has previously been shown for AZD4547 [11]. Others have also reported similar effects on proliferation by TKI258 [9, 10] and AZD4547 [11], and more recently also by BGJ398 [29]. Additionally, TKI258 has been reported to show antitumor activity in FGFR1- and FGFR2-amplified xenograft models [3, 9], and similar early data of clinical trials are also becoming available [9].

In 2D experiments, the viability of BC cells was maintained at 20% or higher after a 70 h treatment with FGFRis, although their proliferation appeared to be blocked at the corresponding inhibitor concentrations. This result suggests that in 2D cultures, FGFRis do not cause overt apoptosis, although the viability at the endpoint is jointly determined by proliferation and cell death. In our proliferation and in cell viability assays, the cells were growing in full serum containing some FGFs and other growth factors, which could have had an impact on the maintenance of viability. In contrast, the numbers of dead cells were increased by FGFRis in 3D MCF-7 and MDA-MB-231(SA) organoids, but not in MFM223 organoids. These differences in sensitivity to drug-induced cell death between 2D and 3D cultures may be due to a prolonged treatment time (3 vs 7 days) or to embedding of the cells in laminin-rich basement membrane extracts like Matrigel, which has been shown to increase the sensitivity to another FGFRi, PD173074 [12]. Our results in the 3D culture system differ from those of Dittmer and co-workers. They did not observe major effects on MCF-7 and MDA-MB-231 cell viability even at a 10 μM TKI258 concentration [30], although similar to our findings, they showed reduced phosphorylation of ERK1/2 in MCF-7 cells and no such effects in MDA-MB-231 cells [30]. The setup of 3D cultures in those experiments was, however, different from that of our organoid cultures and the TKI258 treatment time (3 days) was shorter, which may explain the result differences.

In our 3D cultures, we observed a change in the morphology of the FGFRi-treated organoids, which exhibited a more irregular shape and more multicellular protrusions. This change was most pronounced in MDA-MB-231(SA) organoids treated with low doses of inhibitors. This irregular morphology often indicates poorly differentiated organoids that lack strong cell-cell contacts and/or a basement membrane, or in some cases, increased invasion potential. Invasion and migration were found to be differentially affected in MDA-MB-231(SA) cells. The selective FGFRis BGJ398 and AZD4547 had no effect on migration, but they inhibited invasion of the cells, suggesting that invasion but not migration was dependent on FGFR-mediated mechanisms in these cells. Our observations are in line with the results of Zang et al., who have previously reported TKI258 inhibition of invasion of MDA-MB-231 cells, although they did not study ERK1/2 phosphorylation [31].

The efficacy of FGFRis may be enhanced further by combining them with other compounds or widely used therapeutic approaches. We have previously reported that activation of the FGFR pathway may increase the entry of cells into the G2-M phase [32] and increase proteins involved in M-phase checkpoint control [33]. G2-M is the most radiosensitive phase of the cell cycle [34–36] and, therefore, we hypothesized that FGFRis might increase the radio-sensitivity of BC cells. In addition, the capacity of FGFRis to inhibit proliferation, but not markedly induce apoptosis, suggested that these inhibitors may complement other treatment modalities. Others have reported promising results by combining irradiation with other treatment modalities such as, for example, miRNA treatment in BC [37], FGFRi JNJ-42756493 treatment in rectal cancer [38] and FGFRi PD173074 treatment in squamous cell lung cancer [39]. In our experimental setup, we found that FGFRis did, however, not cause a statistically significant sensitization of the cells to irradiation. FGFRi treatment after irradiation did, however, enhance the effect of irradiation at low concentrations in MFM223 cells and at higher concentrations in MCF-7 cells. These results suggest that, although these FGFRis did not sensitize BC cells to irradiation, they decreased tumor cell repopulation after irradiation and, thus, may have contributed to beneficial treatment outcomes after radiotherapy. In these experiments, the selective FGFRis appeared to be more effective in potentiating the effects of irradiation than the non-selective FGFRi TKI258.

In clinical trials, a 6-fold copy number of *FGFR1* and a 4-fold copy number of *FGFR2* have been used as inclusion criteria for an expected high FGFR expression status, and these patients typically showed responses to FGFRi treatment as indicated by decreased tumor growth [9, 21]. In our study, qRT-PCR analyses of non-selected primary BC samples collected for ex vivo explant cultures did not show increased FGFR mRNA levels compared to the peritumoral tissues from the same patients. When treated in explant cultures with FGFRis, these BC tissues showed, however, a tendency towards decreased proliferation. This finding suggests that even BCs without markedly increased FGFR levels may benefit from FGFRi treatment. Interestingly, we found that FGFRis also seemed to increase the relative number of apoptotic cells. At the tumor tissue level, FGFRis most probably affect not only cancer cells but also tumor-stromal interactions regulated by FGF/FGFR-mediated pathways that may add to BC tissue responsiveness. In breast tumors, FGF/FGFR signaling is also known to contribute to the maintenance of capillary networks and angiogenesis, further increasing FGFRi targets.

Taken together, we conclude that our results suggest that besides targeted therapies of *FGFR*- amplified BC with selective FGFRis, further studies on combination therapy approaches are warranted to obtain efficacious responses to proliferation, migration, invasion and cell death at lower, tolerable concentrations of selective or non-selective FGFRis in BCs with a moderate level of FGFR expression.

## Supplementary Information

ESM 1 (PDF 1.31 mb
