# Supplementary material for: Effects of FGFR inhibitors TKI258, BGJ398 and AZD4547 on breast cancer cells in 2D, 3D and tissue explant cultures
Source: Cell Oncol (Dordr). 2020 Oct 29;44(1):205–18. doi: 10.1007/s13402-020-00562-0 (PMC7907049; doi:10.1007/s13402-020-00562-0)

Supplementary Files to:

Effects of FGFR inhibitors TKI258, BGJ398 and AZD4547 on breast cancer cells in 2D, 3D and tissue explant cultures

Kähkönen TE[1], Toriseva M[1, 2], Petruk N[1, 2], Virta A-R[1], Maher A[1], Eigéliené N[1], Kaivola J[1], Boström P[3], Koskivuo I[4], Nees M[1, 5], Tuomela JM[1, 2], Ivaska KK[1] and Härkönen PL[1 2,]

[1] University of Turku, Institute of Biomedicine, 20520 Turku, Finland
[2] FICAN West Cancer Centre, Turku, 20520 Turku, Finland
[3] Turku University Hospital, Department of Pathology, 20520 Turku, Finland
[4] Turku University Hospital, Department of Plastic and General Surgery, 20520 Turku, Finland
[5] Uniwersytet Medyczny w Lublinie, Dept. of Biomedicine and Molecular Biology II, 20-095 Lublin, Poland

Corresponding author:
Pirkko Härkönen, Institute of Biomedicine, University of Turku, Kiinamyllynkatu 10, 20520 Turku, Finland, Email: harkonen@utu.fi

Supplementary Table S1.

FGFRis used in the study. The IC50 values (nM) for the non-selective FGFRi TKI258 and the FGFR selective inhibitors BGJ398 and AZD4547. The dash symbol (‘ – ‘) refers to no binding to the receptor. The IC50 values are summarized in the following publications: [5] Katoh 2016, [7] Katoh and Nagama 2014, [20] Babina and Turner 2017.

Inhibitor	IC50 (nM)					References
	FGFR1	FGFR2	FGFR3	FGFR4	Other targets	
**TKI258**	8	40	9	-	VEGFR1 (10 nM), VEGFR2 (13 nM), VEGFR3 (8 nM), Kit (2 nM), CSF1R (36 nM), FLT3 (1 nM), PDGFRA (~200 nM), PDGFRB (27 nM)	Katoh and Nagama 2014 Katoh 2016 Babina and Turner 2017
**BGJ398**	0.9	1.4	1	60	-	Katoh 2016
**AZD4547**	0.2	1.8	2.5	165	VEGFR2 (24 nM), Kit (24 nM), CSF1R (9.7 nM), FLT3 (85 nM), IGFR (518 nM)	Katoh and Nagama 2014 Katoh 2016 Babina and Turner 2017

Supplementary Table S2.

Primer sequences and information about primary and secondary antibodies used in the study.

Gene	Forward primer sequence	Reverse primer sequence
FGFR1	5'-TGG CAC CCG AGG CAT TAT TT-3'	5'-CAT GTA CAG CTG GTT GTT GC-3'
FGFR2	5'-AAC AGT CAT CCT GTG CCG AA-3'	5'-AGC CGA AAC TGT TAC CTG TC-3'
FGFR3	5'-CGT CCA CCG ACG AGT ACC T-3'	5'-CTC ACA TTG TTG GGG ACC AGT-3'
FGFR4	5'-CTG ACA CAG TGC TCG ACC TT-3'	5'-AAC CCT GAC ATT TGG GCC AT-3'
beta-actin	5'-CGT GGG GCG CCC CAG' GCA CCA-3	5'-TTG GCC TTG GGG TTC AGG GGG-3'

Antibody	Manufacturer
phospho-FRS2	Cell Signaling Technologies, #3864
phospho-ERK1/2	Cell Signaling Technologies, #4370
total-ERK1/2	Cell Signaling Technologies, #9101
alpha-tubulin	Abcam, ab4074
Anti-rabbit 800CW	Li-Cor, 925-32213
Anti-mouse 680RD	Li-Cor, 925-68072
HRP-conjugated anti-rabbit	Abcam, ab6721
Anti-FGFR1	Abcam, ab10646
Anti-FGFR2	Abcam, ab10648
Anti-FGFR3	Santa Cruz, sc-13121
Anti-FGFR4	Santa Cruz, sc-124

Supplementary Table S3.

Patient information and tumor characteristics. Classification of the tumor samples by a pathologist. The data includes artificial patient ID, age, cancer type and grade, lymph node status, observed metastasis (yes/no), and ER (%), PR (%), HER2 (yes/no) expression, and results from Ki67 staining (%) at tumor dissection by a clinical pathologist.

	**Tumor classification**				**Expression in tumor**			
**Patient ID**	**Type**	**Gradus**	**Nodes**	**Metastasis**	**ER (%)**	**PR (%)**	**HER2**	**Ki67 (%)**
**1**	Ductal	3	0	No	70	95	No	65
**2**	Ductal	3	2/50	Yes	90	90	No	9
**3**	Ductal	2	0/12	No	99	95	No	30
**4**	Lobular	2	29/35	Yes	98	98	No	30
**5**	Ductal	2	3/27	Yes	95	20	Yes	9
**6**	Ductal	2	3/15	Yes	90	98	Yes	30
**7**	Ductal	2	3/8	Yes	98	98	No	30
**8**	Lobular	2	0/8	No	99	95	No	15
**9**	Lobular	2	0/8	No	95	45	No	29
**10**	Lobular	2	0/8	No	98	95	No	12

Supplementary Figure S1.

MCF-7, MDA-MB-231(SA), and MFM223 cells treated with TKI258, BGJ398, and AZD4547. MCF-7, MDA-MB-231(SA) and MFM223 cells were treated with TKI258, BGJ398 and AZD4547 at the indicated concentrations for 70 h. IncuCyte ZOOM microscope imaging was used to monitor cell proliferation in real-time and representative images at the 70 h point are shown.

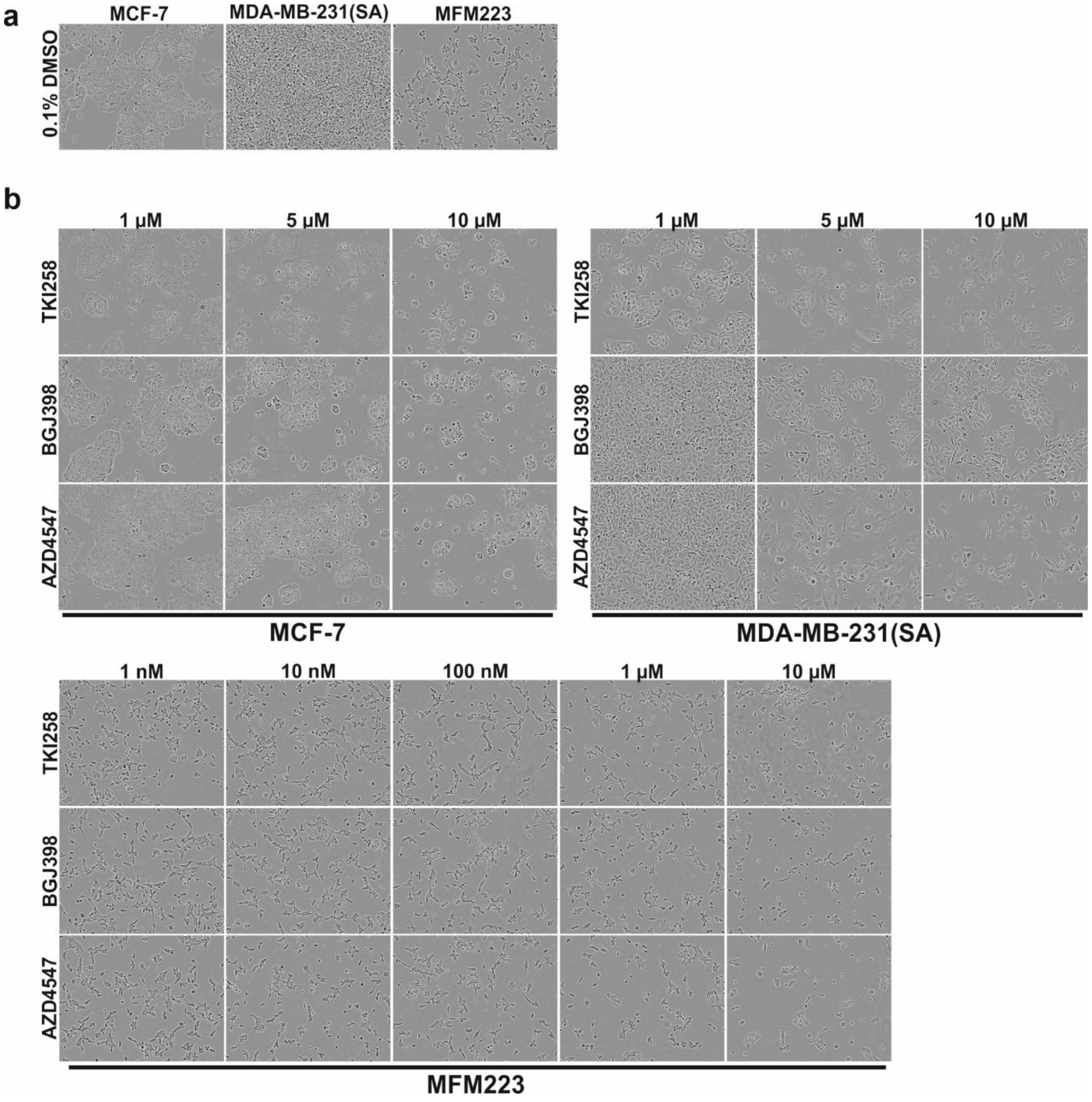

Supplementary Figure S2.

Combination of FGFRis with irradiation. MCF-7 or MDA-MB-231(SA) cells (2000/well) were treated with 1 or 5 µM, and MFM223 cells (10000/well) with 10 nM or 1 µM of FGFRis for 24 h and then irradiated with 0 or 4 Gy X-ray. Medium was changed to FGFRi-free medium after 24 h, and cell viability measured 48 h later (left). Alternatively, cells were first irradiated with 0 or 4 Gy of X-ray and the treatment with FGFRis was started 48 h after irradiation. Medium was changed to FGFRi-free medium after 24 h, and cell viability measured 48h later (right). Cell viability (% of untreated control, mean ± SD, n=3-4) after each treatment in non-irradiated (0 Gy, white bars) and irradiated (4 Gy, black bars) cells. Cells exposed to both FGFRi and irradiation (black) were compared to the vehicle-treated cells exposed to irradiation alone (gray bars, dotted line). Statistical significances are shown for One-Way ANOVA, with Dunnett's test for multiple comparison ($p<0.05$*, $p<0.01$**, $p<0.001$***).

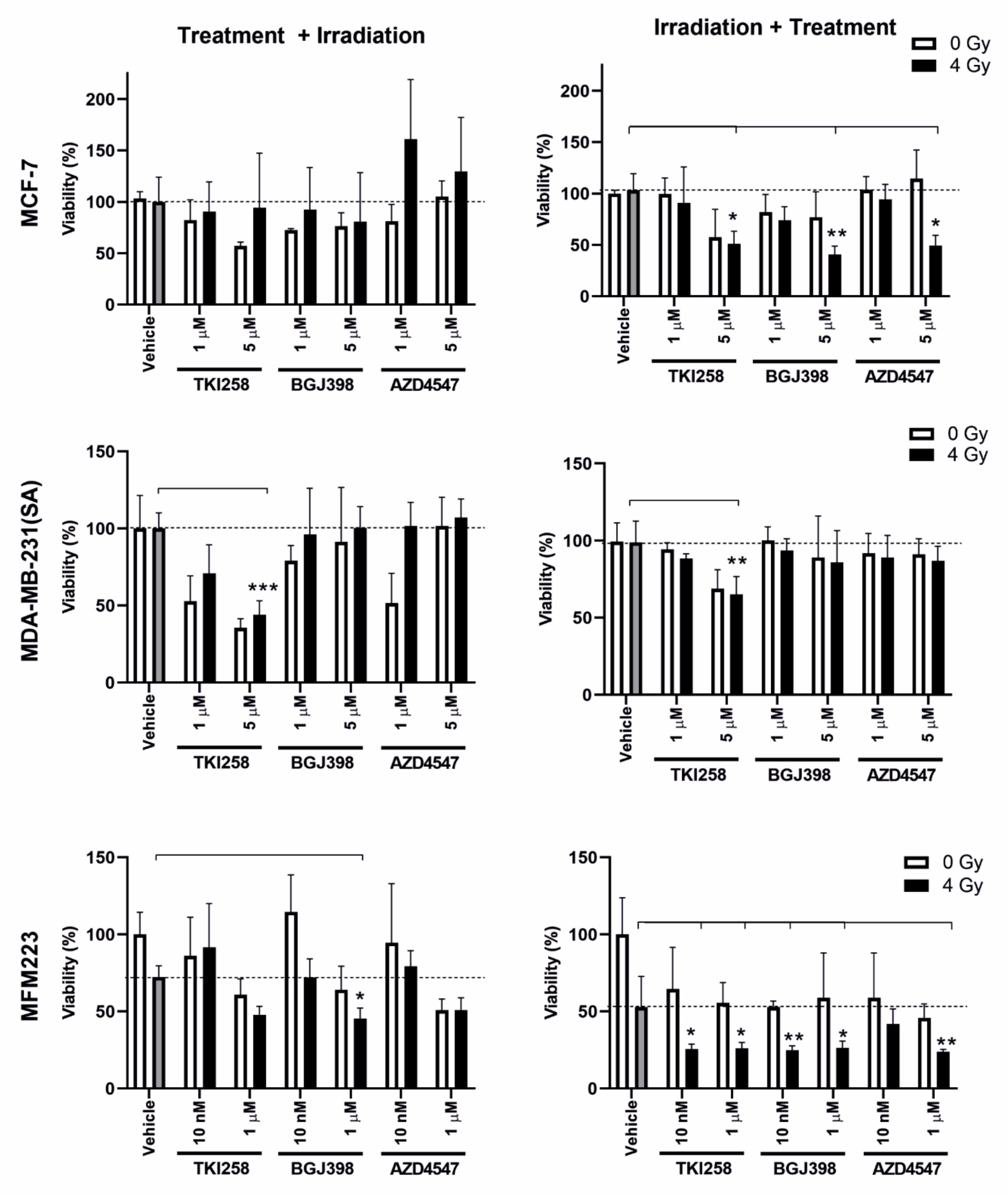

Supplementary Figure S3.

Confocal imaging of organotypic 3D cultures and the morphometric image analysis. A) At the endpoint of cultures, organotypic 3D cultures were stained with Calcein-AM (green) and EthD1 (red) to visualize live and dead cells in organoids, respectively. The organoids were imaged with a confocal microscope with 5x objective, and representative images are shown. B) Maximum projections were segmented and analyzed using AMIDA-software. Example images of the segmentations are shown.

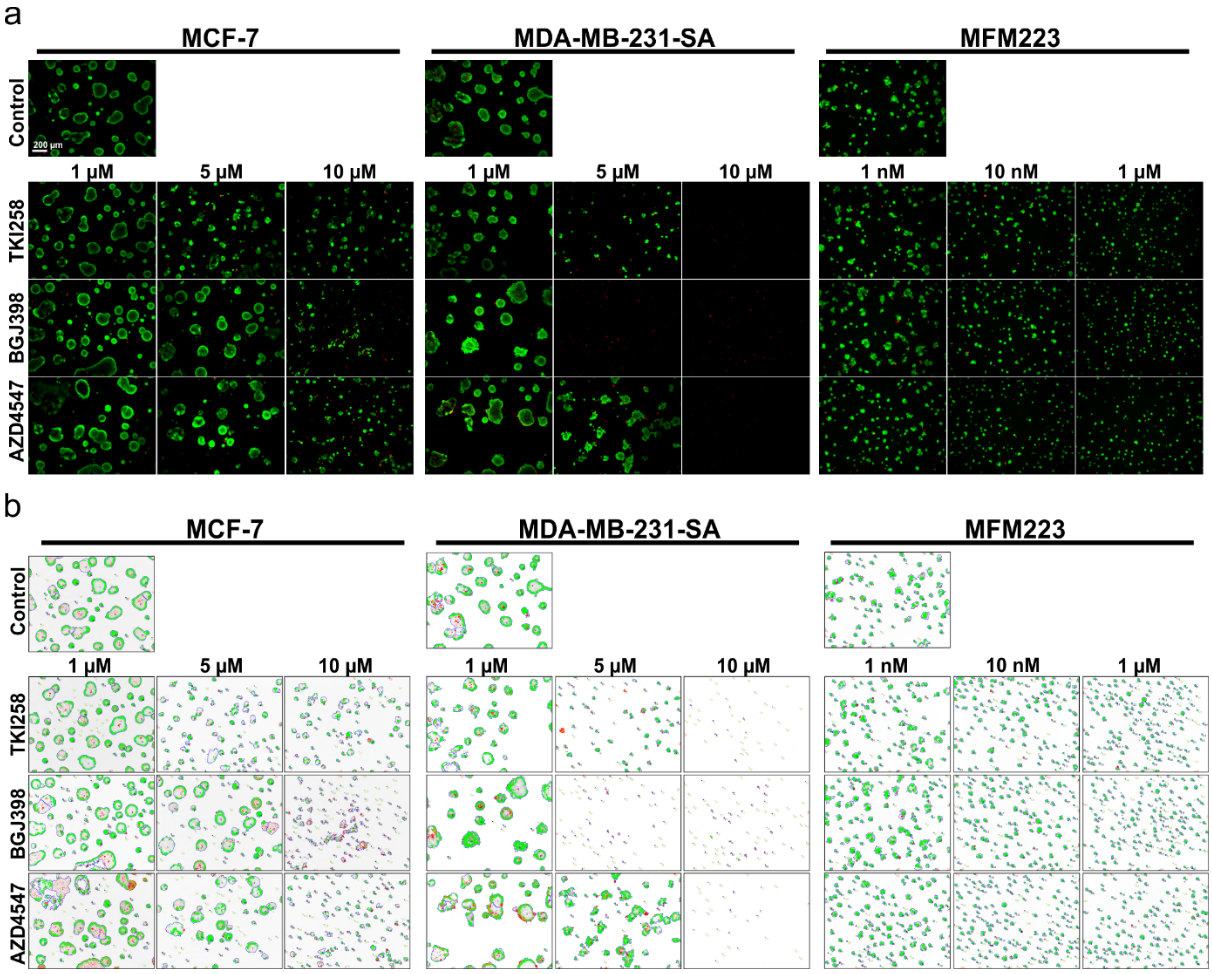

Supplement: Supplementary file 1 — (PDF 1.31 mb [file 13402_2020_562_MOESM1_ESM.pdf]
